# Revolutionizing at‐home testing of blood‐based *p*‐tau217 for early detection of Alzheimer's disease: A validation study of the Tasso Lancet Device

**DOI:** 10.1002/alz70856_102264

**Published:** 2025-12-25

**Authors:** Elisabeth Thijssen

**Affiliations:** ^1^ Neurogen Biomarking, Chicago, IL, USA

## Abstract

**Background:**

P‐tau217 is emerging as the most accurate blood‐based biomarker (BBBM) for the diagnosis of Alzheimer's disease (AD) pathology, with elevated levels correlating with β‐amyloid and tau pathology, as confirmed by PET scans and cerebrospinal fluid analysis. Longitudinal increases in *p*‐tau217 correlate with cognitive decline, making it a promising tool for early detection. Validating remote sampling methods is essential to increase and simplify access to BBBMs for individuals with cognitive impairment.

Neurogen Biomarking provides an end‐to‐end ecosystem for early AD detection, including at‐home sampling, remote cognitive assessments, and access to neurologists. A proof‐of‐concept (POC) study (*N* = 17) showed that *p*‐tau217 concentrations from samples collected with the Tasso+™ device correlated with venipuncture samples and distinguished AD patients from controls. This study aims to validate these findings in a larger cohort.

**Method:**

This validation study includes 70 healthy volunteers and 60 amyloid‐positive AD patients recruited from multiple hospitals, including Insight Hospital, Memorial Health, Pittsburgh University, Banner Health, and Wake Forest University. Blood samples will be collected using the Tasso+™ device and standard venipuncture.

Sample stability will be tested at room temperature (RT) for 24, 36, and 48 hours and samples will be shipped overnight, mimicking real‐world conditions. Samples will be processed using a proprietary method. *p*‐tau217 concentrations will be measured using the ALZpath assay on Simoa^Ⓡ^ technology (Quanterix) and compared between sample types.

**Result:**

Recruitment is ongoing. Based on POC findings, a positive correlation between *p*‐tau217 concentrations from Tasso+™ and venipuncture is expected, analyzed via Spearman's rank correlation. Receiver Operating Characteristic analysis will assess differentiation accuracy between control and amyloid‐positive participants. The AUC values must meet the threshold set by CEOi guidelines for successful validation. The Youden index will determine the optimal *p*‐tau217 cut‐off for amyloid‐PET positivity. The validation results from this study will be first presented at AAIC2025, offering insights into Tasso+™ as a scalable, non‐invasive method for at‐home blood sampling in Alzheimer's biomarker research.

**Conclusion:**

This study aims to validate a novel, non‐invasive at‐home sampling method for *p*‐tau217, facilitating early AD detection and more frequent testing. By improving accessibility, this approach could enhance clinical practice and research, making early diagnosis feasible for a broader population.

